# Hypermethylation of the DPYD promoter region is not a major predictor of severe toxicity in 5-fluorouracil based chemotherapy

**DOI:** 10.1186/1756-9966-27-54

**Published:** 2008-10-20

**Authors:** Ursula Amstutz, Simone Farese, Stefan Aebi, Carlo R Largiadèr

**Affiliations:** 1 Institute of Clinical Chemistry, Inselspital, Bern University Hospital, and University of Bern, CH-3010 Bern, Switzerland; 2 Department of Medical Oncology, Inselspital, Bern University Hospital, and University of Bern, CH-3010 Bern, Switzerland

## Abstract

**Background:**

The activity of dihydropyrimidine dehydrogenase (DPD), the key enzyme of pyrimidine catabolism, is thought to be an important determinant for the occurrence of severe toxic reactions to 5-fluorouracil (5-FU), which is one of the most commonly prescribed chemotherapeutic agents for the treatment of solid cancers. Genetic variation in the DPD gene (DPYD) has been proposed as a main factor for variation in DPD activity in the population. However, only a small proportion of severe toxicities in 5-FU based chemotherapy can be explained with such rare deleterious DPYD mutations resulting in severe enzyme deficiencies. Recently, hypermethylation of the DPYD promoter region has been proposed as an alternative mechanism for DPD deficiency and thus as a major cause of severe 5-FU toxicity.

**Methods:**

Here, the prognostic significance of this epigenetic marker with respect to severe 5-FU toxicity was assessed in 27 cancer patients receiving 5-FU based chemotherapy, including 17 patients experiencing severe toxic side effects following drug administration, none of which were carriers of a known deleterious DPYD mutation, and ten control patients. The methylation status of the DPYD promoter region in peripheral blood mononuclear cells was evaluated by analysing for each patient between 19 and 30 different clones of a PCR-amplified 209 base pair fragment of the bisulfite-modified DPYD promoter region. The fragments were sequenced to detect bisulfite-induced, methylation-dependent sequence differences.

**Results:**

No evidence of DPYD promoter methylation was observed in any of the investigated patient samples, whereas in a control experiment, as little as 10% methylated genomic DNA could be detected.

**Conclusion:**

Our results indicate that DYPD promoter hypermethylation is not of major importance as a prognostic factor for severe toxicity in 5-FU based chemotherapy.

## Background

5-fluorouracil (5-FU) has been one of the most commonly prescribed chemotherapeutic agents in the treatment of various types of cancer for over 40 years with approximately two million patients treated worldwide each year [1]. However, 10–30% of patients receiving the drug develop a severe to life-threatening toxic reaction [2]. 5-FU is a prodrug, thus requiring intracellular conversion into cytotoxic metabolites for anti-tumour effects to take place and giving 5-FU metabolising enzymes a crucial role in determining the effect of the drug [1]. Since about 85% of the administered dose of 5-FU are usually rapidly eliminated, the catabolic pathway, and its key enzyme dihydropyrimidine dehydrogenase (DPD), are particularly important in determining a patient's response to 5-FU [3].

DPD activity is highly variable in the population, with an estimated proportion of 3–5% of individuals showing low or deficient DPD activity [4], which is thought to result in an increased half-life of 5-FU and therefore an increased risk of toxicity [1]. Part of this variability is explained by genetic variation in the DPD gene (DPYD), where over 40 polymorphisms have been described so far. Several of these gene variants have been associated with reduced enzyme activity and severe toxic side effects to 5-FU [1,5-9], the most prominent being a point mutation in the splice site of intron 14 (IVS14+1G>A) resulting in the deletion of exon 14 and thus a non-functional enzyme [10,11]. However, the clinical consequences of this and other mutations in the DPD gene remain unclear, and a large proportion of the observed variation in DPD activity is still unexplained. Recently, partial methylation of the DPYD promoter region has been suggested as an alternative mechanism for DPD deficiency and thus also as a basis for 5-FU toxicity [12]. More specifically, in this study, patients and healthy volunteers with low DPD activity displayed a partially methylated DPYD promoter region, whereas no methylation was detected in individuals with normal enzyme activity. Most interestingly, partial DPYD promoter methylation was observed in all DPD deficient individuals not carrying any known inactivating DPYD mutation. These results thus strongly indicated that this epigenetic mechanism might be an important determinant of DPD activity.

Previously [13], we screened the entire coding region of DPYD in 111 cancer patients receiving 5-FU based chemotherapy, including 24 patients with severe toxic side effects (NCI CTCAE grade ≥ 3). However, none of the patients experiencing severe adverse reactions were carriers of a mutation, which has previously been associated with low DPD activity or severe 5-FU toxicity. On the contrary, some of these gene variants, including the IVS14+1G>A splice site mutation, were detected in patients with no or only mild side effects. Since none of the observed grade ≥ 3 toxicities could be explained by known deleterious DPYD mutations in this study population, we aimed at investigating a potential epigenetic effect in the same patients. In the present study, we therefore assessed the methylation status of the DPYD promoter region in a subsample of this study population, in order to determine the prognostic significance of this epigenetic marker with respect to severe 5-FU toxicity.

**Figure 1:**
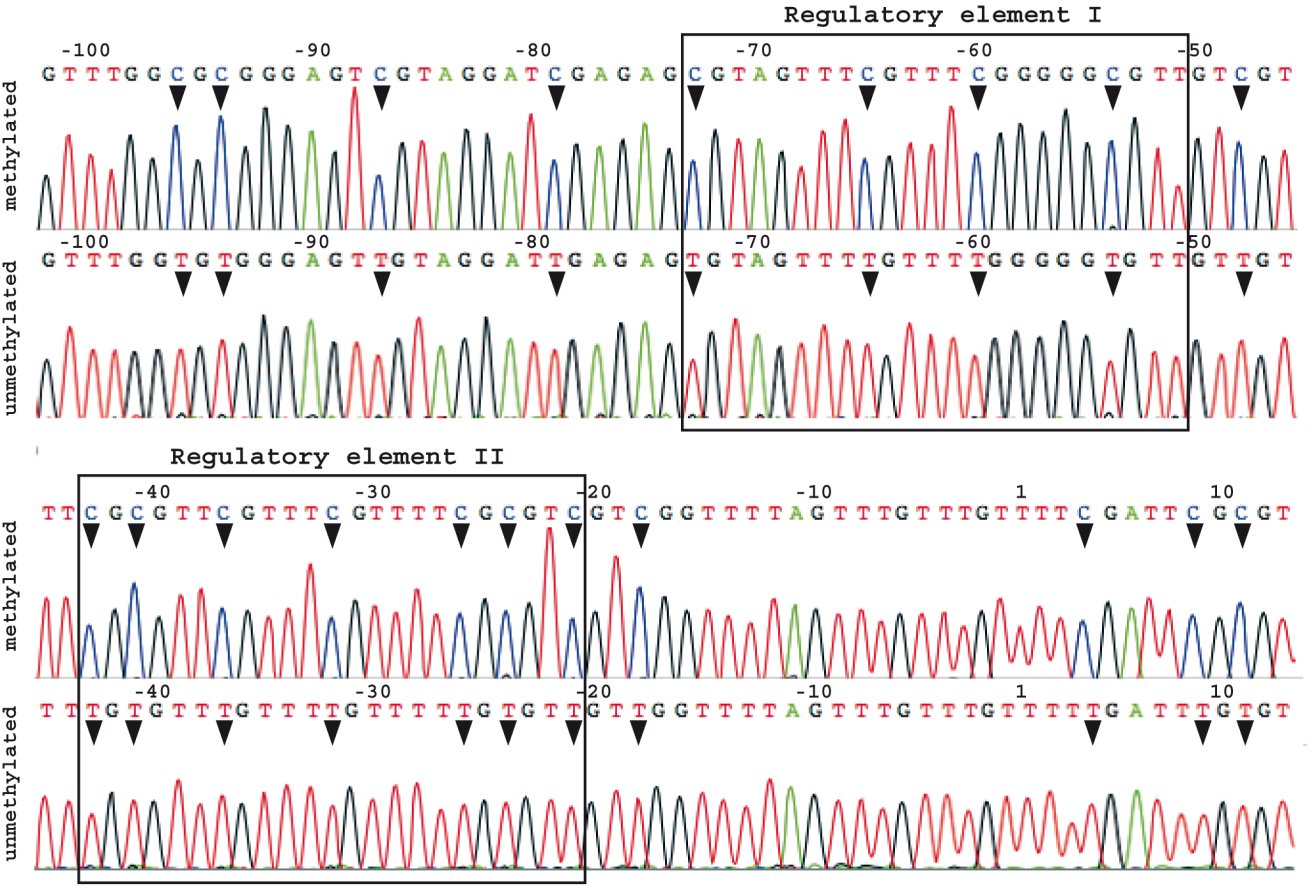
**BSM induced, methylation-specific sequence differences in the DPYD promoter region**. Sequence chromatograms of clone inserts originating from methylated and unmethlyated DNA, obtained in the control experiment using enzymatically methylated DNA. Unmethylated cytosines at CpG sites (indicated with black arrows) are converted to thymines, resulting in a sequence change. The two regulatory elements are shown; numbers indicate the sequence position relative to the transcription start site.

The investigated gene region was a 209 base pair fragment of the 5' untranslated region of DPYD, the same region as investigated in [12]. This fragment contains 27 CpG sites, eleven of which are located within two sequence elements (shown in Figure 1), which have previously been reported to be of regulatory importance [14]. CpG methylation in this region was assessed in peripheral blood mononuclear cells by bisulfite modification (BSM) of genomic DNA, PCR amplification of the specified gene region using methylation-independent primers, and cloning of amplified fragments. Since partial methylation, as it was observed in the previous study [12], would result in a mixture of methylated and unmethylated DNA copies, the cloning procedure enabled us to separately sequence individual amplified fragments, and thus estimate the proportion of methylated DNA in the sample.

## Methods

### Patients

A total of 27 patients were investigated, including 17 patients experiencing severe toxicity following fluoropyrimidine treatment, and ten randomly selected control patients who did not develop any or only a mild to moderate toxic reaction (Table 1). All patients suffered from solid malignant tumors and were treated with various chemotherapy regimens including monotherapies with 5-FU or capecitabine, and combinations with other cytotoxic drugs (Table 1). None of the analysed patients with severe toxic side effects were carriers of a known deleterious DPYD mutation (Table 1), and all non-synonymous mutations detected in these patients were previously observed at similar frequencies also in patients without severe toxic reaction [15]. Blood samples were collected before or during chemotherapy after getting informed consent from all patients; adverse drug effects during the first and second course of chemotherapy were assessed by detailed chart review, and were classified according to the Common Terminology Criteria for Adverse Events (CTCAE) v3.0 [16]. All toxicities equal to or greater than grade three were considered severe.

**Table 1:** Clinical data of investigated patients, DPYD genotype and number of clones sequenced per patient.

					Toxicity grade				N clones	
							
	Sex	Age	Tumor	Regimen	hematologic	gastrointestinal	dermatologic	DPYD genotype (coding region/splice sites)	Total	Methylated
Patients with severe 5-FU related toxicity										
1	m	53	Gastric	5FU-FA	1	3	0	-	21	0
2	f	70	Breast	CAPE	0	3	0	c.85T>C, C29R; c.496A>G, M166V	20	0
3	f	55	Gastric	CAPE-P	3	0	0	c.1601G>A, S534N	21	0
4	m	74	Colon	5FU-FA-P	4	4	0	-	20	0
5	f	53	Colon	5FU-FA-P	3	1	0	c.496A>G, M166V	21	0
6	m	66	Tonsil	5FU-P	2	3	3	c.85T>C, C29R; 1236G>A, E412E	20	0
7	m	53	Oesophagus	5FU-P	4	4	0	-	20	0
8	f	54	Breast	E-5FU-C	3	1	0	1627A>G, I543V	21	0
9	f	65	Gastric	E-5FU-P	3	1	2	-	22	0
10	m	79	Oesophagus	5FU-P	2	3	0	c.85T>C, C29R; c.1236G>A, E412E	20	0
11	f	67	Rectum	5FU-P	4	4	0	c.496A>G, M166V	22	0
12	f	57	Gastric	E-5FU-P	3	1	0	c.85T>C, C29R; c.1236G>A, E412E; c.1627A>G, I543V	20	0
13	m	57	Rectum	5FU	3	0	0	1896T>C, F632F	21	0
14	m	67	unknown^a^	5FU-IRI	3	0	0	c.85T>C, C29R; c.1627A>G, I543V	24	0
15	f	50	Rectum	5FU-FA-P	1	3	0	c.85T>C, C29R	25	0
16	m	75	Gastric	5FU	2	3	0	-	21	0
17	f	47	Breast	CAPE	1	1	3	c.85T>C, C29R; c.496A>G, M166V	30	0

Control patients										
18	f	72	Rectum	5FU	1	1	0	-	20	0
19	f	54	Gastric	5FU-FA-P	2	1	0	c.1679T>G, I560S	21	0
20	m	59	Pancreas	5FU-FA-P	0	1	0	c.85T>C, C29R; c.1627A>G, I543V(H)	20	0
21	m	62	Colon	5FU-FA-P	0	1	0	c.85T>C, C29R (H); c.496A>G, M166V, c.1236G>A, E412E	19	0
22	f	64	Colon	5FU-FA	0	0	0	c.85T>C, C29R; c.1627A>G, I543V	20	0
23	m	63	Oesophagus	5FU-P	1	0	0	-	20	0
24	f	69	Colon	5FU-FA	0	1	0	c.85T>C, C29R (H); c.496A>G, M166V; c.2194G>A, V732I	20	0
25	m	59	Gastric	5FU-FA	1	1	0	c.85T>C, C29R; c.1627A>G, I543V; 1896T>C, F632F	21	0
26	m	59	Colon	5FU-FA-P	1	1	0	c.85T>C, C29R (H); c.496A>G, M166V; c.1627A>G, I543V	20	0
27	m	62	Gastric	E-CAPE-P	2	1	2	c.85T>C, C29R (H); c.496A>G, M166V; c.1236G>A, E412E c.1627A>G, I543V	30	0

Abbreviations: FA, folinic acid; CAPE, capecitabine; P, platinum compound; E, epirubicin; C, cyclophosphamide; H, homozygous carrier ^a ^adenocarcinoma of unknown origin

### Bisulfite modification and PCR amplification of the DPYD promoter region

Genomic DNA was extracted from EDTA-blood samples using the BioRobot EZ1 (Qiagen) and the EZ1 DNA blood 350 μl Kit (Qiagen). Up to 2 μg of genomic DNA were subsequently bisulfite-modified (BSM) using the EpiTect Bisulfite Kit (Qiagen) according to the instructions provided by the manufacturer. A 209 bp fragment of the DPYD promoter region was amplified from BSM samples using a GeneAmp 9700 Thermal Cycler (Applied Biosystems) and the same primers as described in [12]. The 25 μl PCR volume contained 1 μl of BSM DNA, 1 μl of each primer (10 μM), 1 μl of dNTP (10 mM each), 3 μl of MgCl_2 _solution (25 mM), 2.5 μl of AmpliTaq Gold Buffer and 0.5 μl of AmpliTaq Gold DNA polymerase (Applied Biosystems). The following reaction conditions were used: denaturation at 96°C for 10 min, followed by 40 cycles of 50 s at 96°C, 50 s at 52°C, and 1 min at 72°C, and a final extension step at 72°C for 10 min. PCR products were subsequently re-amplified for cloning using either1 μl of undiluted PCR product (if PCR band was not visible or very weak after first amplification) or 1 μl of PCR product diluted 1:100 (if PCR band from first amplification was clearly visible) using touchdown PCR. If amplification with a proofreading polymerase was required for the cloning step (StabyCloning kit), this reamplification step was performed using 0.5 μl of Accuprime GC-rich polymerase (Invitrogen) with 5 μl of the provided 5× buffer, and 0.5 μl of each primer (10 μM) in a 25 μl reaction volume. Otherwise, the same reaction mix as described above was used. The reaction conditions for the touchdown PCR were the following: an initial denaturation step of 10 min at 95°C was followed by five times two cycles with annealing temperatures lowered gradually from 62°C to 54°C in steps of 2°C with 50 s at 94°C, 50 s at the respective annealing temperature, and 1 min at 72°C. This touchdown step was followed by 20 cycles with 50 s at 94°C, 50 s at 52°C, and 1 min at 72°C, and a final extension step of 10 min at 72°C.

### Cloning and sequence analysis

After purification using the QIAquick PCR Purification Kit (Qiagen), PCR products were cloned using the StabyCloning kit (Eurogentec) or the QIAGEN PCR cloning kit (Qiagen) according to the instructions given by the manufacturer. For each sample analysed, a total of 19 to 30 different colonies (Table 1) containing the correct insert were amplified using AmpliTaq Gold DNA polymerase (Applied Biosystems) in the same reaction mix as described above and the primers provided by the manufacturer for the Staby Cloning kit, or T7 and M13 for the QIAGEN PCR cloning kit, respectively. Amplified inserts were subsequently sequenced using the same primers, the Big Dye Terminator v3.1 Cycle Sequencing Kit (Applied Biosystems) and an ABI Prism 3130xl Genetic Analyzer (Applied Biosystems). The obtained sequences were analysed for methylation-specific, bisulfite-induced sequence changes using the software Sequencher v.4.7 (Gene Codes Corporation).

### Enzymatic methylation of genomic DNA

To exclude a potential amplification bias during PCR amplification and to establish the detection limit of the cloning procedure, a control experiment was conducted using various amounts of enzymatically methylated genomic DNA. For enzymatic methylation, genomic DNA from two randomly selected patients was methylated using CpG Methylase M.SssI (New England BioLabs). Approximately 1 μg of DNA was incubated for two hours at 37°C with 1 μl of SssI, 5 μl of NEBuffer 2 (New England BioLabs) and 5 μl of S-adenosylmethionine (1.6 mM) in a reaction volume of 50 μl, followed by an inactivation step of 20 min at 65°C. Reactions were subsequently purified using standard phenol-chloroform purification, followed by ethanol precipitation. The precipitated DNA was resolved in 15 μl of the same elution buffer as used for the isolation of genomic DNA from patient samples. The two methylated DNA samples were subsequently pooled and mixed with BSM genomic DNA from an unmethylated patient sample to contain 10%, 20%, 50% and 100% methlyated BSM DNA, and subjected to the same procedure as described for the patient samples.

## Results and discussion

No evidence of DPYD promoter methylation was observed in any of the 17 investigated patients experiencing severe 5-FU toxicity. Also in the ten control patients, no indication for DPYD promoter methylation was found (Table 1). More specifically, in all of the 19 to 30 cloned fragments analysed per patient, a majority (mean: 98%; range: 81% – 100%) of the 27 CpG sites showed BSM-induced conversions of cytosines to thymines, indicative of unmethlyated cytosines (as shown in Figure 1). On the other hand, increasing numbers of clones containing methylated gene copies, indicated by the preservation of cytosines in the sequence (Figure 1), were detected in the control experiment, according to the amount of enzymatically methylated DNA added. In the fragments considered as showing methylation, on average, 90% of the CpG sites were methylated (range: 41 – 100%). In the sample containing 100% methylated DNA, 18 out of 22 analysed clones contained methylation-specific sequences (82%), indicating an efficiency of the enzymatic methylation of about 80%. One methylated gene copy was detected also in the sample containing only 10% methylated BSM DNA (one out of 20 analysed clones). In the samples containing 20% and 50% methylated DNA, intermediate numbers of methylated clones were observed (five out of 21 and five out of 20 clones, respectively). Therefore, we concluded that as little as 10% methylation of the DPYD promoter region should be detectable using the above-described procedure. Also, the results of this control experiment showed no indication for an amplification bias towards unmethylated gene copies.

The occurrence of severe 5-FU related toxicity in the 17 investigated patients was thus not explained by an epigenetic regulation of the DPYD promoter region. Our findings are in agreement with an other recent study, where no evidence for DPYD promoter methylation was detected in 28 patients with grade 4 toxicity following 5-FU administration [15]. To our knowledge, only the study mentioned above and our own have investigated the predictive potential of DPYD promoter methylation in larger samples of 5-FU treated patients, and both studies did not detect any evidence for such an epigenetic downregulation of DPD. However, although no study so far was able to confirm the initial findings by [12], which were based on a very small sample size, various studies nevertheless suggest epigenetic factors as an alternative explanation for the occurrence of severe 5-FU toxicity where no other molecular basis was found in DPYD [17-19].

Therefore, it is important to recognise that our results and those by [15] strongly indicate that DPYD promoter methylation is, if at all, only of minor importance as a predictive factor for severe toxic side effects in 5-FU based chemotherapy. The molecular basis of severe 5-FU toxicity can thus currently not be attributed to known genetic or epigenetic factors in the DPD gene for a majority of the observed cases in this and other studies [15,17,20]. Whereas it can not be excluded that other, yet unknown genetic or epigenetic factors resulting in a reduced DPD activity may be of value for the prediction of 5-FU toxicity, also various polymorphisms in genes other than DPYD have recently been shown to be correlated with the occurrence of severe adverse side effects to 5-FU [21-23]. A promising approach for future investigations could therefore be to expand the focus from investigating isolated genes like DPYD to a more comprehensive view of taking into account genetic variation in the entire biological pathway [24]. Hopefully, such combinatory approaches will finally lead to results, which are both reproducible and translatable into the clinic.

## Conclusion

Our results indicate that DYPD promoter hypermethylation is not of major importance as a prognostic factor for severe toxicity in 5-FU based chemotherapy.

## Competing interests

The authors declare that they have no competing interests.

## Authors' contributions

CRL, UA and SA conceived the study. UA carried out the experiments and drafted the manuscript. SF participated in the collection of patient samples and information on 5-FU related toxicities. All authors contributed to the writing of the manuscript.
